# Plasticizers May Activate Human Hepatic Peroxisome Proliferator-Activated Receptor **α** Less Than That of a Mouse but May Activate Constitutive Androstane Receptor in Liver

**DOI:** 10.1155/2012/201284

**Published:** 2012-06-20

**Authors:** Yuki Ito, Toshiki Nakamura, Yukie Yanagiba, Doni Hikmat Ramdhan, Nozomi Yamagishi, Hisao Naito, Michihiro Kamijima, Frank J. Gonzalez, Tamie Nakajima

**Affiliations:** ^1^Department of Occupational and Environmental Health, Nagoya City University Graduate School of Medical Sciences, Kawasumi 1, Mizuho-cho, Mizuho-ku, Nagoya 467-8601, Japan; ^2^Department of Occupational and Environmental Health, Nagoya University Graduate School of Medicine, Nagoya 466-8550, Japan; ^3^Laboratory of Metabolism, National Cancer Institute, National Institutes of Health, Bethesda, MD 20892, USA

## Abstract

Dibutylphthalate (DBP), di(2-ethylhexyl)phthalate (DEHP), and di(2-ethylhexyl)adipate (DEHA) are used as plasticizers. Their metabolites activate peroxisome proliferator-activated receptor (PPAR) *α*, which may be related to their toxicities. However, species differences in the receptor functions between rodents and human make it difficult to precisely extrapolate their toxicity from animal studies to human. In this paper, we compared the species differences in the activation of mouse and human hepatic PPAR*α* by these plasticizers using wild-type (*mPPARα*) and humanized *PPARα* (*hPPARα*) mice. At 12 weeks old, each genotyped male mouse was classified into three groups, and fed daily for 2 weeks per os with corn oil (vehicle control), 2.5 or 5.0 mmol/kg DBP (696, 1392 mg/kg), DEHP (977, 1953 mg/kg), and DEHA (926, 1853 mg/kg), respectively. Generally, hepatic PPAR*α* of *mPPARα* mice was more strongly activated than that of *hPPARα* mice when several target genes involving *β*-oxidation of fatty acids were evaluated. Interestingly, all plasticizers also activated hepatic constitutive androstane receptor (CAR) more in *hPPARα* mice than in *mPPARα* mice. Taken together, these plasticizers activated mouse and human hepatic PPAR*α* as well as CAR. The activation of PPAR*α* was stronger in *mPPARα* mice than in *hPPARα* mice, while the opposite was true of CAR.

## 1. Introduction

Dibutylphthalate (DBP), di(2-ethylhexyl)phthalate (DEHP), and di(2-ethylhexyl)adipate (DEHA) are used as representative industrial plasticizers, though the use of the first two considerably decreased recently. These chemicals are involved in peroxisome proliferations, similar to endogenous fatty acids, exogenous fibrates, and thiazolidinediones [1–4]. Once most plasticizers are taken into the body, they are metabolized by lipase in several organs such as liver and small intestine, and their metabolites, especially mono-carboxylic acids, activate peroxisome proliferator-activated receptor alpha (PPAR*α*), and influence the receptor-related lipid metabolism, anti-inflammation, glucose metabolism, and ketogenesis [5].

Peroxisome proliferators (PPs) cause hepatocarcinogenesis in rodents, and PPAR*α* is involved in the mode of action [6]. However, the lower expression of PPAR*α* in human liver [7] and ligand affinity for the agonists [2, 3] has been discussed within the context of how the risk of these chemicals is extrapolated to human from the animal data [8]. Indeed, the International Agency for Research on Cancer downgraded the DEHP carcinogenicity potential from 2B to 3, which produced some conflicting views over the past decade [9–13], but then restored the potential to the 2B grade in 2011 [14]. In addition, recent results showed that not only mouse but also human PPAR*α* was eventually activated by several activators, such as trichloroacetic acid [15] or perfluorooctanoic acid [16], with species differences in PPAR*α*-related gene activation [17]. These results further complicated the risk assessment of peroxisome proliferators.

PPAR*α*-humanized (*hPPAR*α**) mice, so-called *hPPAR*α** ^Tet-OFF^, that express human PPAR*α* only in the liver of *PPAR*α**-null mice were recently established [18]. This mouse line expresses human PPAR*α* considerably higher than mouse PPAR*α* in wild-type mice and is a useful tool to elucidate the former function: 0.1, 0.3 mg/kg b.w. of ammonium perfluorooctanoate-activated mouse PPAR*α*, but not human PPAR*α*, suggesting that the activation of the latter may be weaker than the former [16]. In contrast, when 0.1% Wy-14,643 (which is estimated at about 100 ~ 130 mg/kg b.w.) was administered to wild-type and *hPPAR*α** mice, the functional activations of the target genes such as mitochondrial and peroxisomal **β**-oxidation enzymes were almost the same or slightly less in the latter than in the former [18–20]. Taken together, the activation of human PPAR*α* may be weaker than that of mouse PPAR*α*. However, it is doubtful whether the findings are always similar to the other peroxisome proliferators such as DEHP.

 Constitutive androstane receptor (CAR) is a representative transcriptional regulator for drug-metabolizing enzymes such as cytochrome P450 (CYP), UDP-glucuronosyl transferase (UGT), or sulfotransferase and activated by xenobiotic ligand phenobarbital (PB) or 1,4-bis [2-(3,5-dichloropyridyloxyl)] benzene (TCPOBOP) [21–23]. Many peroxisome proliferators such as DEHP [24] or PFOA [25] are also xenobiotic ligands or activators. On the other hand, CAR plays an important role in lipid homeostasis because of the interactive action with PPAR*α* and inhibition of PPAR*α*-related oxidation of fatty acids [26]. Indeed, TCPOBOP treatment increased serum triglyceride (TG) [27] because of downregulation of *β*-oxidation and upregulation of fatty acid synthesis. However, there is no report whether other phthalates such as DBP and adipates activate CAR and influence lipid homeostasis. It is important to examine whether these phthalates act on CAR because CAR activation is related with liver toxicity, such as modulation of acetaminophen-induced hepatotoxicity [28] or PB-induced liver tumor development [29, 30].

In this study, we selected three plasticizers currently used worldwide, DBP, DEHP, and DEHA, to determine the differences among hepatic mouse and human PPAR*α* and CAR activation in response to these plasticizers using two PPAR*α* mouse lines, wild-type (*mPPAR*α**) and *hPPAR*α** mice. We also investigated how both receptor activations influence plasma and liver TG levels for detection of functional changes in hepatic PPAR*α* and CAR by treatment of plasticizers.

## 2. Materials and Methods

### 2.1. Chemicals

Standard grades of DEHP (≥99.5%), DEHA (≥99.0%), and DBP (≥99.5%) were purchased from Wako Pure Chemical Industries (Osaka, Japan).

### 2.2. Experimental Animals

This study was conducted according to the Guidelines for Animal Experiments of The Nagoya University Animal Center. Two genotyped male mice with a Sv/129 genetic background, *hPPARα* [18] and wild-type *mPPAR*α**, were used to identify respective PPAR*α* functions in the lipid metabolism. All mice were housed in a temperature- and light-controlled environment (25°C, 12 h light/dark cycle) and maintained on stock rodent chow and tap water *ad libitum*. At 12 weeks old, each genotyped mouse was classified into three groups: one group was treated with corn oil daily for two weeks by gavage (vehicle control group); the other two were treated with 2.5 or 5.0 mmol/kg DEHP (977, 1953 mg/kg), DEHA (926, 1853 mg/kg), or DBP (696, 1392 mg/kg), for two weeks. No significant differences were observed in the body weight at the start of the three plasticizer treatments (data not shown). On the next day after the last dose (18–20 hours later), all the mice were killed by decapitation, and the blood and livers were removed. The liver samples were stored at −80°C until use; as for the blood, after centrifuging at 3,500 g for 10 min, the plasma was stored at −80°C until use.

### 2.3. Nuclear Fraction

A nuclear fraction was extracted from a part of the frozen liver using a CelLytic NuCLEAR Extraction Kit (SIGMA, Tokyo, Japan).

### 2.4. Analysis of Protein Concentrations

Each tissue was homogenized with a three-fold volume of 10 mM phosphate buffer (pH 7.4) containing 0.25 M sucrose. Protein concentrations of the homogenate samples were measured using a Protein Assay Kit (Bio-Rad, Tokyo, Japan).

### 2.5. Lipid Concentrations in Plasma and Liver

Lipid from liver was extracted using the method of Folch et al. [31]. TG in the liver and plasma measured using a TG-IE kit (Wako, Osaka, Japan).

### 2.6. Histopathological Analysis

The organs fixed in 10% neutral buffered formalin were embedded in paraffin and sliced into 2 *μ*m sections. Tissue sections of the livers were stained with hematoxylin and eosin and examined under a light microscope using the BZ-8000 (Keyence Corporation, Osaka, Japan). Histopathological findings were scored according to the degree of lipid accumulation and necrosis with inflammatory cell infiltration.

### 2.7. Real-Time Quantitative PCR

Total RNA was isolated using RNeasy Mini Kit (QIAGEN, Tokyo, Japan). Complementary DNA (cDNA) was synthesized from 1 *μ*g of total RNA using Oligo(dT)_20_ primer. RNA quantity and quality were checked by a GeneQuant II RNA/DNA Calculator (Pharmacia Biotech, Framingham, MA). Primers were designed using Primer Express software (Applied Biosystems) based on the sequence of the respective GI number, as shown in the Supplemental Table available online at doi:10.1155/2012/201284. As for MTP and Cyp4a14, primers were used elsewhere [26, 32]. These mRNA levels were monitored by the ABI PRISM 7000 Sequence Detection system (Applied Biosystems, Foster City, CA), as described previously [16, 33, 34].

### 2.8. Western Blotting

Western blotting was conducted by the method described previously [35]. Briefly, the samples for electrophoresis adjusted to 10 *μ*g protein in liver homogenates of nuclear fraction were subjected to 10% SDS-PAGE and transferred to the nitrocellulose membranes. After blocking with 3% skim milk, each membrane was incubated with the primary antibody, followed by incubation with alkaline phosphatase-conjugated goat anti-rabbit IgG (Jackson Immuno Research, West Grove, PA). The primary polyclonal antibody was prepared using purified medium-chain acyl-CoA dehydrogenase (MCAD) [36], keto-acyl-CoA thiolase (PT) [37], very long-chain acyl-CoA dehydrogenase (VLCAD) [38], and peroxisomal bifunctional protein (PH) [39]. These antibodies were already used elsewhere [15]. The primary polyclonal antibodies of PPAR*α* were purchased from Santa Cruz Biotechnology, Inc. (CA). Each band was quantified using densitometry, the Lane & Spot Analyzer version 5.0 (ATTO Corporation, Tokyo, Japan) as described elsewhere [16, 33, 35]. Each band was normalized to the respective level of glyceraldehyde-3-phosphate dehydrogenase.

### 2.9. Electrophoretic Mobility Shift Assay (EMSA)

The following oligonucleotides, synthesized by Sigma Aldrich Japan (Tokyo, Japan), were used as probes based on the sequence of DR-4 nuclear-receptor-(NR-) binding sites reported by Kim et al. [40]: NR-1 probe, 5′-biotin-TCTGTACTTTCCTGACCTT-3′; NR-2 probe, 5′-biotin-TCAACTTGACTGACACC-3′.LightShift Chemiluminescent EMSA kit (Pierce Biotechnology, Rockford) was used with a slight modification. Sample mixture contained nuclear extract (4 *μ*g), 0.2 mg/mL poly (dI-dC), 5% glycerol, 0.1% NP-40, 5 mM MgCl_2_, 0.2 mM EDTA, 2% Ficol (400), 47 mg/mL transfer RNA, and 2 *μ*M biotin-labeled double-stranded oligonucleotide. The reaction samples were resolved on nondenaturing electrophoresis (4% acrylamide) and transferred to a positively charged nylon membrane (Roche Diagnostics, Mannheim, Germany). Constitutive androstane receptor (CAR)-NR-1 and CAR-NR-2 complexes were detected with a Chemiluminescent Nucleic Acid Detection Module (Pierce Biotechnology) and visualized using a Lumi Vision PRO HS II (Aisin Seiki Co., Ltd., Japan).

### 2.10. Statistical Analysis

Comparisons were made using the two-way analysis of variance (ANOVA) and the Tukey-Kramer HSD post hoc test. A logarithmic transformation was applied to MTP-mRNA before statistical analysis. Values of *P* < 0.05 were considered to indicate statistical significance.

## 3. Results

### 3.1. Body and Liver Weights

No significant differences were observed in body weight after the treatments (Table 1). Exposure to 2.5 (low-dose) and 5.0 mmnol/kg (high-dose) DEHP and DEHA increased both liver weight and liver/body weight ratio only in *mPPAR*α** mice, but high-dose DBP increased only the absolute liver weights (Table 1). In contrast, treatment with any plasticizer failed to influence either the liver weight or the liver/body ratio in* hPPAR*α** mice.

### 3.2. TG in the Plasma and Liver

The plasma TG level in *mPPAR*α** control mice was similar to that in *hPPAR*α** controls (Table 1). High-dose DEHA increased plasma TG levels in *hPPAR*α** mice, but not in *mPPAR*α** mice. In contrast, the other plasticizers did not influence the levels. In each of the control mice, hepatic TG levels were significantly greater in *hPPAR*α** mice than in the *mPPAR*α** mice (Table 1). High-dose DEHP and DEHA decreased the levels in the liver of *mPPAR*α** mice. High-dose DEHP increased the levels in *hPPAR*α* mice*, whereas DEHA did not. DBP did not influence the TG levels in both genotyped mice. Thus, the TG decrease due to the accelerated lipid metabolism was seen in *mPPAR*α** mice treated with DEHP or DEHA. In contrast, hepatic TG accumulation was seen in DEHP-treated *hPPAR*α** mice.

### 3.3. Histopathological Changes

**Figure 1:**
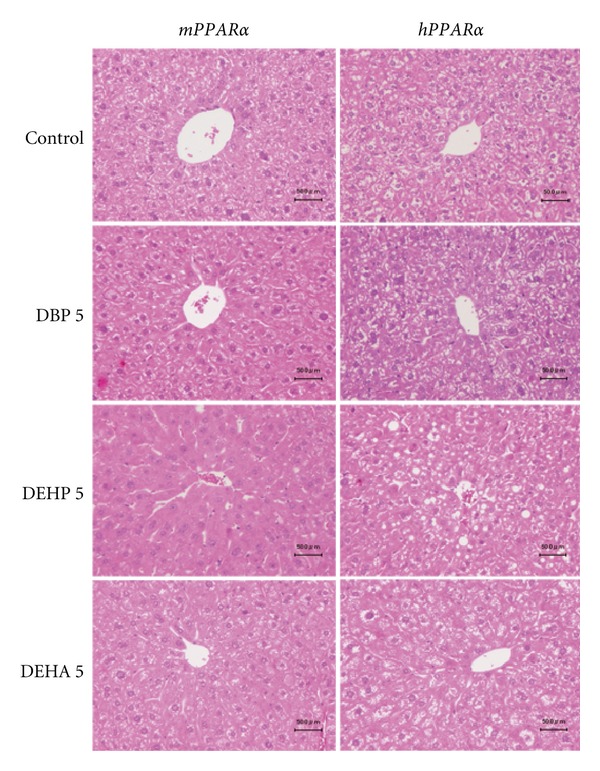
Histopathological changes in livers in *mPPAR*α** and *hPPAR*α** mice treated with control, high-dose DBP, DEHP, and DEHP for 2 weeks. Hepatocellular enlargements were prominently observed in *mPPAR*α** mice of DEHP group and slightly in those of DEHA and DBP. Moderate eosinophilic cytoplasm was observed in *mPPAR*α** mice treated with DEHP. Cytoplasmic vacuoles due to lipid accumulation were seen in *hPPAR*α** mice exposed to three plasticizers. Each scale bar indicates 50 *μ*m.

In the control animals, no obvious differences in the scores of lipid accumulation, inflammatory and necrotic cell infiltrations were observed in the liver between both genotyped mice (Figure 1, scores not shown). As mentioned above, hepatic TG levels were greater in *hPPAR*α** controls than *mPPAR*α** controls; however no obvious histopathological differences in lipid accumulation were found between the two genotyped mice. The hepatocellular enlargements were prominently observed in *mPPAR*α** mice of the high-dose DEHP group and slightly in those of high-dose DEHA and DBP groups. Cytoplasmic vacuoles due to lipid accumulation were seen in *hPPAR*α** mice exposed to the three plasticizers, though the changes were not dose dependent. A focal necrosis with inflammatory cells was seen in two of five *hPPAR*α** mice exposed to high-dose DEHP, all animals exposed to high-dose DEHA and three of five animals exposed to low-dose DEHA. Moderate eosinophilic cytoplasm which may result from the increase in peroxisome or mitochondria was observed in all *mPPAR*α** mice treated with high-dose DEHP; however, the finding was minimal in those on the low dose. In contrast, only two of five animals on high-dose DBP and DEHA exhibited minimal or mild eosinophilic cytoplasm, respectively. Taken together, popular histopathological changes caused by peroxisome proliferators such as liver enlargement and eosinophilic cytoplasm were prominent in* mPPAR*α** mice treated with high-dose DEHP. On the other hand, focal necrosis was seen mainly in *hPPAR*α** mice exposed to high-dose DEHA.

### 3.4. PPAR*α* and Target Genes

**Figure 2:**
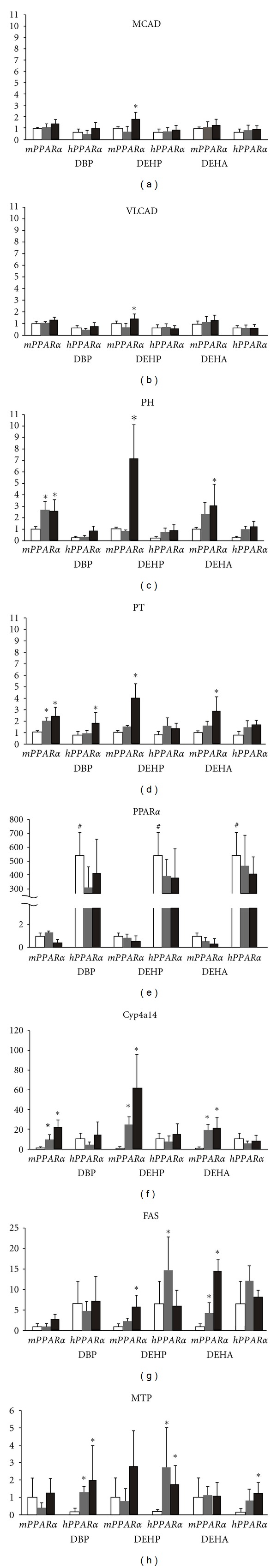
mRNA expressions of hepatic PPAR*α* and its related genes in duplicate analyses. Expressions of mRNA were analyzed by quantitative real-time PCR. Each mRNA was normalized to the level of GAPDH-mRNA expression in the same preparation, and mean of control in *mPPAR*α** mice was assigned a value of 1.0. White, gray, and black columns represent control values, 2.5 mM- and 5.0 mM-treated group, respectively. Each column and bar represents mean ± S.D., respectively. A logarithmic transformation was applied to MTP-mRNA before statistical analysis. *Significantly different from respective controls (*P* < 0.05). ^#^Significantly different among genotypes (*P* < 0.05).

Low-dose DBP significantly increased PH- and PT-mRNA levels (2.7-fold and 2.0-fold, resp.) in *mPPAR*α** mice (Figure 2), whereas low-dose DEHP and DEHA did not. In high-dose groups, all plasticizers increased hepatic peroxisomal PH- and PT-mRNA in *mPPAR*α** mice, while DBP alone induced PT-mRNA in *hPPAR*α** mice. The increases were greatest in DEHP-treated *mPPAR*α** mice (7.1-fold and 4.1-fold, resp.), and those by DBP and DEHA treatments were almost the same (2.6-fold, 2.5-fold and 3.0-fold, 2.9-fold, resp.). All plasticizers at low dose did not influence hepatic mitochondrial MCAD- and VLCAD-mRNA levels. High-dose DEHP, however, increased both mRNA levels only in *mPPAR*α** mice, but only marginally (1.8-fold and 1.4-fold, resp.).

**Figure 3:**
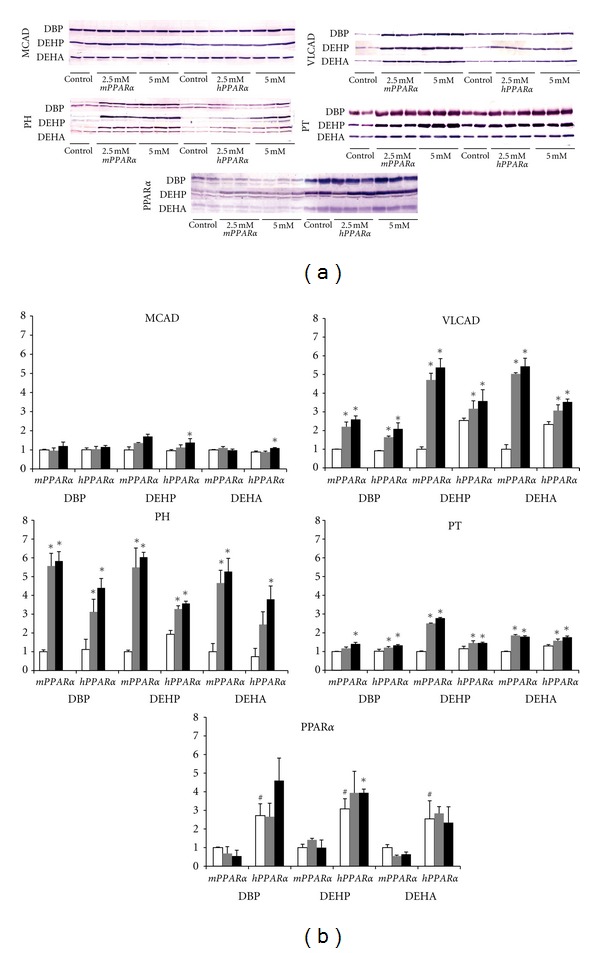
(a) Western blotting analysis of hepatic PPAR*α* and related genes. All mice from each treatment and genotype were examined across two gels, one of which is shown here. (b) Western blotting analysis of hepatic PPAR*α* and related genes. Each band was quantified by densitometric analysis as described in Materials and Methods, and mean strength of control in *mPPAR*α** mice was assigned a value of 1.0. White, gray, and black columns represent control values, 2.5 mM- and 5.0 mM-treated group, respectively. Each column and bar represents mean ± S.D., respectively. *Significantly different from respective controls (*P* < 0.05). ^#^Significantly different among genotypes (*P* < 0.05).

All plasticizers at low dose increased PH and PT protein in the liver of both genotyped mice except PH in DEHA-treated *hPPAR*α** mice and PT in DBP-treated* mPPAR*α** mice (Figures 3(a) and 3(b)). All plasticizers at high dose also increased PH and PT protein in the livers of both *mPPAR*α** and *hPPAR*α** mice. The inductions of PH were slightly stronger in *mPPAR*α** exposed to DBP and DEHP (DBP, 5.9-fold; DEHP, 6.0-fold; DEHA, 5.3-fold) than in *hPPAR*α** mice (3.9-fold, 1.9-fold, 5.1-fold, resp.). The increases of PT by DEHP or DEHA treatments were also stronger in *mPPAR*α** (2.8-fold and 1.8-fold, resp.) than in *hPPAR*α** mice (1.3-fold and 1.4-fold, resp.), although those by DBP were almost the same in both *mPPAR*α** and *hPPAR*α** mice.

In mitochondrial enzymes, three plasticizers at any doses increased hepatic VLCAD protein expressions in both *mPPAR*α** and *hPPAR*α** mice. The inductions appeared to be stronger in *mPPAR*α** mice exposed to DEHP and DEHA (DBP: 2.6-fold, DEHP: 5.4-fold, DEHA: 5.4-fold) than in corresponding *hPPAR*α** mice (2.3-fold, 1.4-fold, 1.5-fold, resp.), similar to peroxisomal enzyme PH. High-dose DEHP and DEHA increased hepatic MCAD levels in *mPPAR*α** and *hPPAR*α** mice, and in *hPPAR*α** mice, respectively, whereas DBP did not affect the levels in either *mPPAR*α** mice or *hPPAR*α** mice.

Low- and high-dose DEHA, DEHP, and DBP also increased hepatic Cyp4a14, a microsomal enzyme involved in *ω*-oxidation of many plasticizers, expressions only in *mPPAR*α** mice but not in *hPPAR*α** mice (Figure 2). Inductions in the former mice were 23-fold, 62-fold, and 21-fold at high-dose DBP, DEHP, and DEHA, respectively.

In the control group, the expression of PPAR*α* was significantly greater in *hPPAR*α** mice than in *mPPAR*α** mice either in the mRNA (540-fold) or protein (about 3-fold) levels (Figures 2, 3(a), and 3(b)). No treatments elevated mouse and human PPAR*α*-mRNAs. High-dose DEHP increased only PPAR*α* protein expression in *hPPAR*α* mice*, but other treatments did not. 

Low- and high-dose DEHA and high-dose DEHP significantly increased FAS-mRNA to 4.4-fold and 14.7-fold, and 5.8-fold in *mPPAR*α** mice, respectively (Figure 2). Low-dose DEHP also increased it to 14.9-fold in *hPPAR*α** mice. However, DBP treatment did not influence FAS-mRNA in both genotype mice. We also measured MTP-mRNA levels in the liver: low- and high-dose DBP and DEHP increased the mRNA to 8.8-fold and 13.5-fold, and 18.8-fold and 11.8-fold, respectively, in *hPPAR*α** mice but not in *mPPAR*α** mice. Similarly, high-dose DEHA increased MTP-mRNAs (8.5-fold) only in *hPPAR*α** mice.

Collectively, inductions of peroxisomal, mitochondrial, and microsomal enzymes involved in *β*-oxidation were stronger in *mPPAR*α** mice than in *hPPAR*α** mice treated with plasticizers in terms of mRNA levels, whereas transporter enzyme was induced only in *hPPAR*α** mice exposed to plasticizers.

### 3.5. CAR and Target Gene

**Figure 4:**
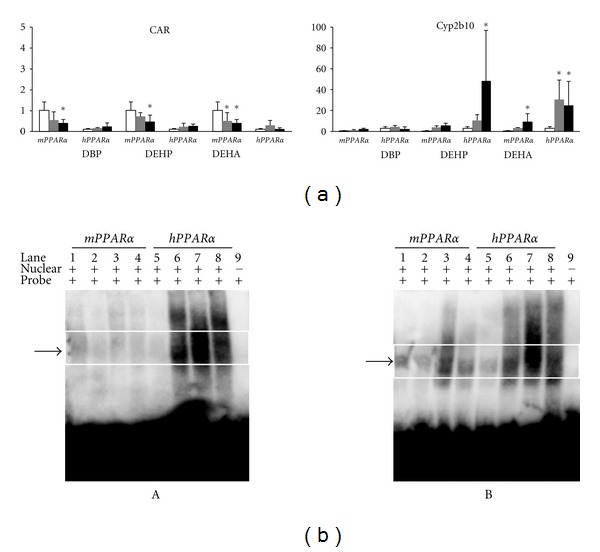
(a) Effects on hepatic expressions of CAR and Cyp2b10-mRNA levels. Each mRNA level was normalized to the level of GAPDH mRNA in the same preparation, and the mean of the control group in wild-type (*mPPAR*α**) mice was assigned a value of 1.0. White, gray and black columns represent control values, 2.5 mM- and 5.0 mM-treated group, respectively. Values are expressed as mean ± S.D. *Significantly different from respective control group (*P* < 0.05). (b) Electrophoresis mobility shift assays of CAR-NR-1 (A) and CAR-NR-2 (B) complexes in liver nuclear fraction from control or treated-*mPPAR*α** (wild-type) and *hPPAR*α** mice. Lanes 1 and 5, control of wild-type, respectively; lanes 2 and 6, wild-type and *hPPAR*α** mice treated with 5.0 mM DBP, respectively; lanes 3 and lane 7, wild-type and *hPPAR*α** mice treated with 5.0 mM DEHP, respectively; lanes 4 and lane 8, wild-type and *hPPAR*α** mice treated with 5.0 mM DEHA, respectively; lane 9, oligonucleotide for NR-1 or NR-2 only. Arrows indicate the shifted CAR-NR complex.

Low- and high-dose DEHA and high-dose DEHP and DBP decreased CAR-mRNA levels in *mPPAR*α** mice, but the levels in *hPPAR*α** mice were not affected at any dose (Figure 4(a)). In contrast, high-dose DEHP strongly induced typical CAR target gene, Cyp2b10-mRNA, in *hPPAR*α** mice (48.3-fold). Low- and high-dose DEHA induced Cyp2b10-mRNA levels in *hPPAR*α** mice (31.2-fold and 24.5-fold, resp.). The high-dose DEHA also elevated the mRNA levels in *mPPAR*α** mice (9.2-fold), but only marginally compared with those in *hPPAR*α** mice. In contrast, DBP did not influence the levels in both genotyped mice.

The treatments with all plasticizers dramatically induced NR-1 (Figure 4(b) A) and NR-2 (Figure 4(b) B) DNA-binding activity of hepatic CAR in *hPPAR*α** mice at high dose. The high-dose DEHP also induced NR-2-binding activity in *mPPAR*α** mice, but DBP or DEHA did not. The activities in *hPPAR*α** mice were strongest in the DEHP-treated group, followed by the DEHA- and DBP-treated group.

In summary, plasticizers, especially in DEHP or DEHA, bind to hepatic CAR and markedly induce CAR-target gene mainly in *hPPAR*α** mice.

## 4. Discussion

The present study clearly shows that three plasticizers (DEHP, DEHA, and DBP) significantly activated mouse hepatic PPAR*α* in *mPPAR*α** mice, but the activation of human hepatic PPAR*α* in *hPPAR*α** mice was weaker than that of the former mouse line even at the high-dose exposure, especially in peroxisomal *β*- or *ω*-oxidation. Among the three plasticizers, DEHP is the strongest from the standpoint of PPAR*α*-mediated gene responses. These results are consistent with *in vitro* studies [3, 4] which demonstrated that mono (2-ethylhexyl) phthalate (MEHP) activated mouse PPAR*α* at lower concentrations and exhibited a stronger response than those of human PPAR*α* [4], and MEHP activated mouse and human PPAR*α* at a lower concentration than the respective monoesters of DBP and DEHA [3, 4]. Interestingly, these species differences in PPAR*α* activation were most prominent in microsomal PPAR*α*-target gene, Cyp4a14, followed by mitochondrial (MCAD, VLCAD) or peroxisomal enzymes (PH, PT). Notably, all the plasticizers also activated CAR preferentially in *hPPAR*α** mice. The activation was also stronger in DEHP than DEHA judging from the target gene (Cyp2b10) as well as the DNA-binding (NR-1 and 2) activity analysis.

As mentioned above, DEHP and DEHA activated PPAR*α* and CAR preferentially in *mPPAR*α** and *hPPAR*α** mice, respectively. Our finding is very similar to the fact that DEHP induced Cyp2b10 more strongly in the livers of *PPAR*α**-null mice than* mPPAR*α**ones [24, 41]. Although the reason why CAR induction was stronger in *hPPAR*α** mice than in *mPPAR*α** mice remains unclear, it is likely that CAR is more easily activated when the function of PPAR*α* is weak, as with human PPAR*α* in* hPPARa *mice [15] or lack of PPAR*α* in *Ppar*α**-null mice [41]. CAR was reported to crosstalk with PPAR*α* and suppress its related gene expressions such as Cyp4a14 and carnitine palmitoyltransferase 1*α* in the liver of mice [26, 27]. It is of interest that DEHP activated both receptors more than DEHA. However, the chemical form of the activator for each receptor may be different; since MEHP did not induce Cyp2b10 in JWZ-CAR cell line [42], the parent substance itself may be an activator of CAR. No report on DEHA indicated that either the parent substance itself or the metabolite(s) is a preferential activator for CAR. In the present study, DBP also induced binding activity of CAR in *hPPAR*α** mice but did not increase Cyp2b10-mRNA in that strain, though DBP has been reported to activate CAR in the liver of rats [43]. Interestingly, the CAR2 splice variant of human CAR is activated by DEHP [44], which suggests that human CAR may also play an important role in DEHP toxicity. Taken together, CAR-mediated effects by plasticizers should be noted as a novel aspect of their toxicities to provide a new rationale to evaluate toxicity correctly.

Species differences of mouse and human PPAR*α* activation by Wy-14,643 have been investigated using *mPPAR*α** and *hPPAR*α** mice fed 0.1% Wy-14,643-containing feed for 2 weeks *ad libitum* [18], at a dose roughly estimated to be 0.3 ~ 0.4 mmol/kg/day. This dose significantly induced peroxisomal and mitochondrial fatty acid-metabolizing enzymes such as acyl-CoA oxidase, VLCAD, and MCAD, followed by a similar decrease in serum triglycerides in both mouse lines. Even a lower dose of Wy-14,643 than the plasticizers used in this study was presumed to activate mouse and human PPAR*α* to a similar extent along with decreased plasma TG levels. This result suggests that there may not be a species difference in the activation by Wy-14,643. Since all plasticizers induced PPAR*α*-related enzymes involved in *β*- or *ω*-oxidation in *mPPAR*α** mice but none of them influenced the plasma TG level, the PPAR*α* activation by Wy-14,643 is not coincident with the present study from the standpoint of PPAR*α*-target gene induction as well as plasma TG levels.

DEHP was the strongest inducer of PPAR*α*-related *β*-oxidation enzymes in *mPPAR*α** mice among the three chemicals. It was also the strongest activator for CAR in both* mPPAR*α**and *hPPAR*α** mice in our study. However, Wy-14,643 did not activate CAR [41]. In this regard, the effect of Wy-14,643 on the nuclear receptors is different from that of DEHP. TCPOBOP, a CAR potent agonist, was suggested to cause an accumulation of serum TG [26, 27], whereas the PPAR*α* agonist Wy-14,643 decreased it. These opposite actions by CAR and PPAR*α* in TG homeostasis [45] may reflect the plasma TG unchanged by DEHP, because DEHP induced both PPAR*α* and CAR. In contrast, the *hPPAR*α** mice exposed to high-dose DEHA had elevated plasma TG. In these mice, MTP-mRNA, which was involved in the transport of TG from liver to blood, was induced and may partly be the reason for the increased plasma TG, even though CAR was also induced by DEHA treatment.

As for TG levels in livers, the high dose of DEHP or DEHA decreased the levels in *mPPAR*α** mice, whereas DEHP increased the levels in *hPPAR*α** mice. The increase in *hPPAR*α** mice, as different from that in *mPPAR*α** mice, may be ascribed to the weaker inductions of enzymes involved in *β*- and *ω*-oxidation in *hPPAR*α** mice than in *mPPAR*α** mice. MEHP increased TG in hepatocyte culture of guinea pig because of the weak induction of *β*-oxidation and lauric acid hydroxylation, whereas it decreased TG in rat hepatocytes due to the significant induction of these enzymes [46]. The degree of *β*-oxidation-related enzyme inductions by DEHP was comparable between mice and rats [34]. Taken together, the difference in mouse and human PPPAR*α* functions presumably produced the different effects of DEHP or DEHA on hepatic TG accumulation between *mPPAR*α**and* hPPAR*α** mice.

In the present study, we only investigated the effects of three kinds of plasticizers on the lipid metabolism and did not investigate DEHP- or DEHA-caused tumors in relation to PPAR*α*. CAR is thought to mediate the hepatocarcinogenic effects of xenobiotics [29], suggesting that it may contribute to the PPAR*α*-independent hepatocarcinogenesis observed in *PPAR*α**-null mice following chronic DEHP exposure [35]. DEHP at a 1150 mg/kg dose for 4 days induced CAR and Cyp2b10-mRNAs only in *PPAR*α**-null mice, and 200 mg/kg DEHP induced them in both wild-type and *PPAR*α**-null mice [41]. The induced rate was greater in the latter than the former mice, suggesting that *PPAR*α**-null mice are more susceptible to DEHP-induced CAR signaling compared to that of *mPPAR*α** mice. DEHP activated not only PPAR*α* but also CAR, though Wy-14,643 did not activate CAR [41]. This different signaling suggests that the molecular mechanism of carcinogenicity in phthalates may not always be the same as that of Wy-14,643.

Finally, hepatic mRNAs of cell cycle-related genes such as cyclin D1, protooncogene such as c-jun, and apoptosis-related gene Bax, were measured using *mPPAR*α** and *hPPAR*α** mice exposed to the plasticizers, but these mRNA levels did not increase in both genotyped mice; instead, decreases of cell cycle-related genes were observed in both genotyped mice (unpublished data), which is not consistent with the case of Wy-14,643 [19]. These results again suggest that DEHP-induced molecular signalings are not always the same as those by Wy-14,643. The reason for this is unclear, but the weaker affinity of DBP, DEHP, and DEHA for human and mouse PPAR*α* than Wy-14,643 may be a possible explanation [4].

In conclusion, these plasticizers activated not only mouse and human hepatic PPAR*α* but also CAR, and the activation of PPAR*α* was stronger in *mPPAR*α** mice than in *hPPAR*α** mice, while that of CAR was the opposite. Thus, DEHP is not only a PPAR*α* agonist but also a CAR activator, which may trigger each function.

## Supplementary Material

Primer lists.

## Figures and Tables

**Table 1:** Body, liver weights and TG levels after treatment with plasticizers for 2 weeks.

		B.W.	Liver weight	Liver weight/ B.W. (%)	Plasma TG	Liver TG
*mPPar*α**	Control	23.9 ± 0.91	0.88 ± 0.11	3.68 ± 0.38	79.4 ± 16.3	14.8 ± 1.53
	DBP 2.5	25.9 ± 2.05	1.08 ± 0.13	4.14 ± 0.17	89.9 ± 24.8	12.5 ± 2.76
	DBP 5.0	26.7 ± 2.01	1.20 ± 0.10*	4.49 ± 0.40	113.9 ± 40.4	11.4 ± 1.68
	DEHP 2.5	22.1 ± 1.82	1.13 ± 0.11*	5.09 ± 0.24*	82.6 ± 13.8	11.6 ± 1.56
	DEHP 5.0	22.9 ± 0.92	1.26 ± 0.06*	5.54 ± 0.33*	84.0 ± 24.5	6.8 ± 0.90*
	DEHA 2.5	25.9 ± 0.85	1.20 ± 0.07*	4.63 ± 0.22*	136.9 ± 15.9	11.4 ± 0.90
	DEHA 5.0	24.2 ± 1.81	1.28 ± 0.18*	5.27 ± 0.35*	119.5 ± 36.3	7.5 ± 1.76*

*hPPar*α**	Control	22.7 ± 2.20	1.04 ± 0.06	4.59 ± 0.25	97.0 ± 23.6	24.4 ± 5.51^#^
	DBP 2.5	25.0 ± 2.32	1.07 ± 0.08	4.29 ± 0.18	127.0 ± 35.0	22.6 ± 4.66
	DBP 5.0	23.1 ± 4.51	1.05 ± 0.28	4.76 ± 0.29	95.1 ± 26.0	31.9 ± 19.31
	DEHP 2.5	23.8 ± 2.58	1.12 ± 0.17	4.69 ± 0.25	111.5 ± 28.0	20.6 ± 4.66
	DEHP 5.0	21.6 ± 2.58	1.03 ± 0.17	4.52 ± 0.37	67.8 ± 35.0	30.9 ± 4.24*
	DEHA 2.5	24.9 ± 1.03	1.12 ± 0.08	4.48 ± 0.14	142.3 ± 59.9	23.1 ± 1.98
	DEHA 5.0	24.7 ± 2.94	1.23 ± 0.17	4.98 ± 0.25	176.0 ± 41.0*	28.4 ± 2.73

B.W: body weight.

Each value represents mean ± S.D. *Significantly different from respective controls (*P* < 0.05). ^#^Significantly different from *mPPAR*α** controls (*P* < 0.05).

## Abbreviations

ANOVA: Analysis of variance
CAR: Constitutive androstane receptor
CV: Central vein
DBP: Dibutylphthalate
DEHP: Di(2-ethylhexyl)phthalate
DEHA: Di(2-ethylhexyl)adipate
DGAT: Diacylglycerol acyltransferase
EMSA: Electrophoretic mobility shift assay
*hPPAR*α**: Humanized PPAR*α* mouse
MCAD: Medium-chain acyl-CoA dehydrogenase
MEHP: Mono(2-ethylhexyl)phthalate
*mPPAR*α**: Wild-type mouse
MTP: Microsomal triacylglycerol transfer protein
NR: DR-4 nuclear receptor binding site
PB: Phenobarbital
PH: Peroxisomal bifunctional protein
PPAR**α**: Peroxisome proliferator-activated receptor *α*
PT: Keto-acyl-CoA thiolase
TG: Triglyceride
VLCAD: Very long-chain acyl-CoA dehydrogenase.
