# Supplementary material for: Plasticizers May Activate Human Hepatic Peroxisome Proliferator-Activated Receptor α Less Than That of a Mouse but May Activate Constitutive Androstane Receptor in Liver
Source: PPAR Res. 2012 Jun 20;2012:201284. doi: 10.1155/2012/201284 (PMC3388330; doi:10.1155/2012/201284)
Supplement: Supplementary file 1 — Primer lists. [file 201284.f1.doc]

Supplemental table Primer lists

|  | GI number | Forward | Reverse |
| --- | --- | --- | --- |
| mPPARα | 7106384 | TTTCCCTGTTTGTGGCTGCTA | CCCTCCTGCAACTTCTCAATG |
| hPPARα | 33875149 | GCGATCTAGAGAGCCCGTTATC | GCCAAAGCTTCCAGAACTATCC |
| MCAD | 6680617 | TTGGCACGTTCTGATCCAGAT | GCTGGCCCATGTTTAATTCCT |
| VLCAD | 23956083 | TGGCTTCAAGGTTGCTGTCA | GATCAACCGCCTTGGCAAT |
| PT | 18700003 | GGATGCTTCCGTGCTGAGAT | TCCTGAGACACGGTGATGGTT |
| PH | 31541814 | TGGGCTGTCACTATCGGATTG | AGAGCAACAGGAACTCCAACGA |
| CAR | 2267575 | CAGGGTTCCAGTACGAGTTTTTG | AGGCTCCTGGAGATGCAGTC |
| Cyp2b10 | 4731349 | TGCCCTTCTCAACAGGACAAA | GGCAATGCTTTCACCAAGACA |
| DGAT1 | 31981804 | GGCGGTCCCCAACCAT | GCTCTGCCACAGCATTGAGA |
| DGAT2 | 118129808 | TGGCCTGCAGTGTCATCCT | TTCCAGTCAAATGCCAGCC |
| GAPDH | 66396585 | AGAACATCATCCCTGCATCCA | CCGTTCAGCTCTGGGATGAC |
